# Tracking oxidation-induced alterations in fibrin clot formation by NMR-based methods

**DOI:** 10.1038/s41598-021-94401-3

**Published:** 2021-08-03

**Authors:** Wai-Hoe Lau, Nathan J. White, Tsin-Wen Yeo, Russell L. Gruen, Konstantin Pervushin

**Affiliations:** 1 Lee Kong Chian School of Medicine, Nanyang Technological University, Singapore, Singapore; 2 Department of Emergency Medicine, University of Washington School of Medicine, Seattle, Washington USA; 3 ANU College of Health and Medicine, The Australian National University, Canberra, ACT Australia; 4 Communicable Diseases Centre, Institute of Infectious Disease and Epidemiology, Tan Tock Seng Hospital, Singapore, Singapore; 5 School of Biological Sciences, Nanyang Technological University, Singapore, Singapore

**Keywords:** Biochemistry, Biological techniques, Biotechnology, Molecular biology

## Abstract

Plasma fibrinogen is an important coagulation factor and susceptible to post-translational modification by oxidants. We have reported impairment of fibrin polymerization after exposure to hypochlorous acid (HOCl) and increased methionine oxidation of fibrinogen in severely injured trauma patients. Molecular dynamics suggests that methionine oxidation poses a mechanistic link between oxidative stress and coagulation through protofibril lateral aggregation by disruption of AαC domain structures. However, experimental evidence explaining how HOCl oxidation impairs fibrinogen structure and function has not been demonstrated. We utilized polymerization studies and two dimensional-nuclear magnetic resonance spectrometry (2D-NMR) to investigate the hypothesis that HOCl oxidation alters fibrinogen conformation and T_2_ relaxation time of water protons in the fibrin gels. We have demonstrated that both HOCl oxidation of purified fibrinogen and addition of HOCl-oxidized fibrinogen to plasma fibrinogen solution disrupted lateral aggregation of protofibrils similarly to competitive inhibition of fibrin polymerization using a recombinant AαC fragment (AαC 419–502). DOSY NMR measurement of fibrinogen protons demonstrated that the diffusion coefficient of fibrinogen increased by 17.4%, suggesting the oxidized fibrinogen was more compact and fast motion in the prefibrillar state. 2D-NMR analysis reflected that water protons existed as bulk water (T_2_) and intermediate water (T_2i_) in the control plasma fibrin. Bulk water T_2_ relaxation time was increased twofold and correlated positively with the level of HOCl oxidation. However, T_2_ relaxation of the oxidized plasma fibrin gels was dominated by intermediate water. Oxidation induced thinner fibers, in which less water is released into the bulk and water fraction in the hydration shell was increased. We have confirmed that T_2_ relaxation is affected by the self-assembly of fibers and stiffness of the plasma fibrin gel. We propose that water protons can serve as an NMR signature to probe oxidative rearrangement of the fibrin clot.

## Introduction

Fibrinogen is a 340 kDa glycoprotein, physiologically present in blood plasma at concentrations from 2 to 4 g/L, that self-associates to form the fibrin clot at wounds after its activation by thrombin^1^. It is composed of two pairs of three non-identical chains Aα, Bβ, and γ connected with 29 disulfide bonds. Together, the chains comprise a symmetrical molecule consisting of one globular E region flanked on each side by globular D regions that are connected by three-stranded alpha-helical coiled coils^2,3^. Fibrin is formed by thrombin-mediated proteolytic cleavage and removal of N-terminal fibrinopeptides from the respective Aα and Bβ chains. The exposed binding sites of α- and β-“knobs” are complementary to a- and b-“holes” in the γ and β nodules in the D regions of adjoining monomers. Knob-hole associations each result in the formation of half-staggered, double-stranded protofibrils, which then associate laterally to form fibrin fibers, and ultimately the branching hydrogel network structure of the hemostatic clot^4^. Lateral association is contributed to by interactions between αC regions of the fibrin monomers (αC-αC interactions) during polymerization, which is important for mechanical stiffness, stability, and durability of fibrin clots^5–7^. Polymerization of fibrinogen lacking its αC regions is delayed but not completely inhibited^7^.

Fibrinogen is also highly susceptible to many post-translational modifications (PTMs), especially from oxidants and free radicals^3^. Oxidant agents are a potentially important source of oxidative fibrinogen PTMs that have been identified in trauma patients having impaired clot formation^13^. Neutrophils are recruited to the blood after trauma and are activated to release histones and DNA as neutrophil extracellular traps (NETs) and myeloperoxidase (MPO), which synthesizes HOCl by the MPO/ hydrogen peroxide (H_2_O_2_) oxidant system^8^. HOCl is predominantly generated in the plasma, where it can oxidize methionine residues to methionine sulfoxide as a host-mediated bacterium killing mechanism^9^. Recent evidence supports an important role for fibrinogen in the regulation of these responses by sequestering histones as histone-fibrinogen complexes^10^. We have previously reported that exposure of fibrinogen to HOCl in vitro has profound effects upon fibrin polymerization, producing weak, soft, and thin fibered fibrin clots that are resistant to enzymatic degradation by the protease plasmin^11^. We found that methionine at positions AαM476, BβM367, and γM78 were oxidized to methionine sulfoxide by HOCl, though the AαC domain was preferentially oxidized^11^. Molecular dynamics (MD) simulation revealed that oxidation within the AαC domain may promote the opening of key beta-hairpin structures making αC domain dimerization energetically unfavorable in comparison with native structures^12,13^. These data suggest a relationship and potential mechanism by which PTMs by oxidants generated in the blood after trauma injury can contribute directly to impaired clot formation. This is potentially significant because the risk of death for trauma patients presenting with impaired clot formation is increased up to sixfold^14^.

NMR is an important and useful tool for assessing the structure of gels by examining the movement of water molecules which can be affected by fibrillar structures, cells, vessels, extracellular matrix, and other macromolecules contained therein^15^. Parameters including spin–lattice (T_1_) relaxation and spin–spin (T_2_) relaxation time and diffusion coefficients can be used to identify important structural characteristics^15,16^. Molecular fingerprints of complex biological fluids such as blood have been encoded using two-dimensional (2D) T_1_/T_2_ correlations. NMR relaxometry has been used to investigate oxygenated (oxy-Hb), deoxygenated (deoxy-Hb), and oxidized (oxidized Hb) hemoglobin (Hb) derivatives with respect to their altered T_1_/T_2_ relaxation states^17^. Spin–spin (T_2_) relaxation times have also been used to probe fibrin clot structure via paramagnetic properties of hemoglobin in erythrocytes entrapped within whole blood clots^18–20^.

In this investigation, we explored HOCl-induced alterations of fibrinogen and fibrin clots using NMR-based methods including a high polarizing magnetic field strength above 600 MHz as well as a benchtop NMR spectrometry operating at 80 MHz. One of our goals was to establish the detection limit of the NMR applications to track blood clot structure alterations which can be associated with pathology in blood clot formation or coagulopathy. We hypothesized that HOCl-induced fibrinogen oxidation alters fibrinogen in the prefibrillar state and alters fibrin gel properties that are detectable using NMR methods. To test this hypothesis, we used 2D-NMR to determine ^1^H relaxation and diffusion properties of fibrinogen solutions and fibrin gels upon oxidation. We reported evidence that T_2_ relaxation of water protons reflects the alternation of fibrinogen conformation and rearrangement of fibers in the fibrin network after oxidation. Since NMR spectrometer operating at 80 MHz is effective, we foresee that NMR-based method can be further developed as a potential diagnostic tool to assess the prospect of coagulopathy via detecting alterations of fibrin clot structure due to oxidation in situ.

## Results

### Oxidation alters fibrin polymerization similarly to inhibition of AαC domain interactions

**Figure 1:**
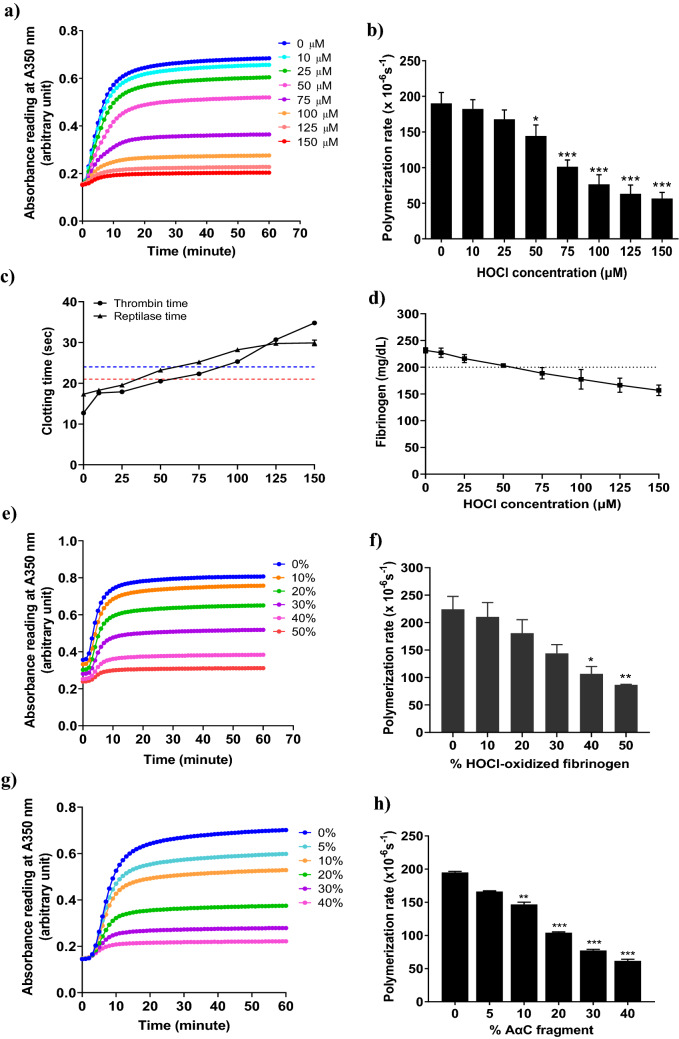
(**a**) Turbidity curves of non-oxidized fibrinogen (0 µM HOCl) and purified fibrinogen solutions oxidized with an increasing HOCl concentrations (10, 25, 50, 75, 100, 125, 150 μM). Fibrinogen without HOCl oxidation (0 µM HOCl) was used as a control. (**b**) Polymerization rate for fibrinogen control and the fibrinogen solutions oxidized with increasing HOCl concentrations (10, 25, 50, 75, 100, 125, 150 μM). (**c**) Thrombin and reptilase times for the fibrinogen control and fibrinogen solutions oxidized with increasing HOCl concentrations. Thrombin and reptilase time measured the clotting time needed for fibrin clot formation after the addition of thrombin and reptilase, receptively. The normal value of thrombin time and reptilase time are < 21 s and < 24 s, respectively. The red and blue dotted lines indicate the upper limit of the thrombin and reptilase times required for normal fibrin clot formation. (**d**) The concentration of functional fibrinogen in fibrinogen solution after HOCl oxidation. Clauss fibrinogen was used to measure the concentration of functional fibrinogen in the fibrinogen control and purified fibrinogen solutions oxidized with an increasing HOCl. The normal fibrinogen level is at the range of 2–4 mg/mL (200–400 mg/dL). The black dotted line indicates the upper limit of normal fibrinogen concentration. (**e**) Turbidity curves of control plasma fibrinogen and plasma fibrinogen solution added with an increasing level of HOCl-oxidized fibrinogen (10, 20, 30, 40, and 50%). Plasma fibrinogen solution without HOCl-oxidized fibrinogen (0% HOCl-oxidized fibrinogen) was used as control. (**f**) Polymerization rate for control plasma fibrinogen and plasma fibrinogen solution added with increasing HOCl-oxidized fibrinogen (pre-oxidation of purified fibrinogen by 150 µM HOCl). (**g**) Turbidity curves of fibrinogen control and the purified fibrinogen solutions added with an increasing percentage of AαC fragment (5, 10, 20, 30, and 40%). The copolymerization of the protein mixtures was activated by incubation with 0.16 NIH U/mL thrombin at 37 °C for 1 h. (**h**) Polymerization rate of fibrinogen control and the purified fibrinogen added with an increasing percentage of AαC fragment.* P* values that were 0.05 and less were considered statistically significant, as assessed using ANOVA-Bonferroni test. * *P* < 0.05; ** *P* < 0.01; *** *P* < 0.001.

Lateral aggregation of growing individual fibrin fibers is accompanied by branching, forming the three-dimensional structure of fibrin network. Oxidation is thought to affect fibrin self-assembly predominantly by inhibiting the lateral association of protofibrils^11,21^. Turbidimetry of fibrin gel is widely used for examining the polymerization events, including the initiation of protofibril aggregation via cleavage of fibrinopeptide by thrombin, the rate of protofibril association, and self-assembly of fibrils to form fibers^22^. Robust fibrin polymerization is characterized by rapid fibrinopeptide cleavage, protofibrils lateral aggregation, and thick fibrin fibers formation. It is represented by the turbidity curve with a short lag time, maximal slope, and high maximal turbidity. Therefore, we chose turbidity assay to explore the effects of HOCl oxidation on fibrin polymerization kinetics. As shown in Fig. 1a, HOCl oxidation impaired fibrin polymerization by increased lag times, decreased maximal slopes and decreased maximal turbidity (Fig. 1a). The polymerization rate for protofibrils association was decreased with HOCl oxidation in a dose-dependent manner (Fig. 1b). The fibrin polymerization rate was significantly altered by 75 µM HOCl oxidation, with a 41% decrease in polymerization rate as compared to fibrinogen control (Fig. 1b). Fibrinogen solution was significantly oxidized by 150 µM HOCl with 70% reduction of polymerization rate (Fig. 1b). The maximal turbidity of the fibrin gels was significantly decreased by HOCl oxidation (Fig. 1a), with the fibrinogen control (0 µM HOCl) forming a very opaque fibrin gel and the HOCl-oxidized fibrinogen (150 µM HOCl) forming a transparent fibrin gel.

We then used thrombin time and reptilase time assays using a steel ball coagulometer to confirm that the turbidity changes were represented an alternation of fibrin clot mechanics and structure rather than inhibition of clotting^23,24^. These assays are used to identify qualitative changes in fibrin polymerization (e.g., dysfibrinogenemia) and our previous studies have demonstrated high sensitivity to the degree of oxidation^21,23^. Thrombin time measures the clotting time required for a fibrin clot to form at 37 °C after addition of thrombin, which activates the conversion of fibrinogen monomers to fibrin by cleavage of fibrinopeptides A and B. Reptilase time uses batroxobin, a viper venom enzyme, as an alternative thrombin to confirm the presence of dysfibrinogenemia and measure the clotting time required for fibrinogen converted to fibrin by specific cleavage of fibrinopeptide A. Unlike thrombin, batroxibin is not sensitive to the effects of heparins. Both thrombin and reptilase times were significantly prolonged by HOCl oxidation at 75 µM and higher concentrations compared to fibrinogen control (Fig. 1c), reflecting HOCl oxidation disrupted polymerization of fibrinogen and impaired fibrin clot structure. Additionally, to add clinical relevance, the Clauss fibrinogen assay was performed to measure the concentration of functional fibrinogen. It is the most common clinical method used for quantification of functional fibrinogen^24^. The functional fibrinogen concentrations were significantly decreased with HOCl oxidation in a dose-dependent manner (Fig. 1d). Again, fibrinogen solutions oxidized by 75 µM, and higher HOCl concentrations induced functional fibrinogen levels below normally acceptable clinical ranges (Fig. 1d).

To confirm that oxidized fibrinogen could also disrupt clot formation in human plasma fibrinogen, we then examined the effects of adding HOCl-oxidized fibrinogen (pre-oxidation of purified fibrinogen solution by 150 µM HOCl) to normal pooled human plasma prior to fibrin clot formation. As a result of HOCl-oxidized fibrinogen, the lag times increased and the maximal slopes, the maximal turbidity (Fig. 1e), and the polymerization rate (Fig. 1f) were decreased. The rate of polymerization for plasma fibrinogen solution was significantly decreased by adding 50% HOCl-oxidized fibrinogen (Fig. 1f). These results confirm that indirect oxidation of plasma fibrinogen solutions by adding HOCl-oxidized fibrinogen disrupted fibrin clot formation in clinically relevant ways.

We then examined the impacts of specifically blocking AαC domain interactions during fibrin polymerization for its similarity to HOCl oxidation by using a recombinant human AαC domain peptide fragment as a competitive inhibitor of AαC domain interactions. The fibrinogen alpha C domains^11^ enhance protofibrils lateral aggregation to form thick fibrin fibers^25,26^. MD simulations suggested that oxidation of AαM476 inhibits fibrin protofibrils lateral aggregation by shifting the equilibrium between the open and closed conformation of the AαC domains in favor of the former^26^. We synthesized a homogeneous recombinant human fibrinogen AαC 419–502 (AαC fragment) corresponding to the human fibrinogen alpha C domain to be used as a specific competitive inhibitor of AαC domain interactions during fibrin polymerization. Protein BLAST analysis reported that the synthesized human AαC fragment shared 61% identity to the bovine sequence (Uniprot ID: P02672) and contained ordered structure at the N-terminal (Supplementary Fig. S1a). The AαC fragment was also confirmed to have one methionine residue at 476 and no tryptophan residues in its amino acid sequence (Supplementary Fig. S1a). Homology modeling of its 3D structure was also aligned with the human fibrinogen alpha chain precursor (Uniprot ID: P02671) as analyzed by I-TASSER^27^. The best-predicted model of human AαC fragment is shown in Supplementary Fig. S1b. The AαC fragment also showed a local concentration of negative charge at the N-terminal sub-domain and Met476 residue was exposed on the surface (Supplementary Fig. S1c). Since the alpha C domain is highly susceptible to proteolysis, we characterized the purity of AαC fragment by SDS-PAGE (Supplementary Fig. S2a) and confirmed its identity by ESI-TOF mass spectrometry analysis (Supplementary Fig. S2b).

We then clotted the purified fibrinogen solutions (native) in the presence of an increasing percentage of the AαC fragment through thrombin activation. The final concentration of fibrinogen was maintained at 0.6 μM (2 mg/mL) at its lower physiological condition. Adding as little as 10% AαC fragment to native fibrinogen significantly decreased the maximal slopes of fibrin polymerization, maximal turbidity (Fig. 1g), and the polymerization rate (Fig. 1h), which were considerably more affected in comparison to lag times (Fig. 1g). The impairment of fibrin clot structure was very similar to those measured in the presence of increasing HOCl oxidation (Fig. 1a vs. g), suggesting that HOCl oxidation and competitive inhibition of AαC domain interactions have similar disruptive effects on fibrin polymerization.

### Oxidation alters fibrinogen structure and function in solution

Fibrinogen monomers are made up of two sets of A*α*, B*β*, and *γ* chains bound by disulfide bonds. As expected, we observed three bands corresponding to A*α* (67 kDa), B*β* (55 kDa), and *γ* (45 kDa) chains of the fibrinogen control on SDS-PAGE. Exposure of fibrinogen solutions to increasing concentrations of HOCl up to 150 μM did not cause distinct changes in the electrophoretic banding pattern under reducing conditions (Supplementary Fig. S2c), suggesting that HOCl-induced oxidation does not fragment or significantly alter the molecular weight of individual fibrinogen chains.

CD spectroscopy was then utilized to investigate secondary structural changes of fibrinogen upon HOCl oxidation. The CD spectra of protein in the far ultraviolet (UV) range (180–250 nm) depends on the electronic excitation of the partially delocalized peptide bonds, which form the backbone of the polypeptide chain^28^. Therefore, changes in the main alpha-helical peptide backbone structure of fibrinogen would be identifiable using this method. The far-UV CD spectra of the control and the oxidized fibrinogen solutions (with increasing HOCl concentrations) showed similar characteristics of α-helical structure, exhibiting double negative bands at 208 and 222 nm (Supplementary Fig. S3a), suggesting that HOCl oxidation of fibrinogen did not alter its overall α-helical backbone structure.

The hydrodynamic properties of fibrinogen solutions are concentration-dependent and largely determined by the intermolecular self-association of fibrinogen molecules mediated by the highly flexible and extensible αC regions^29^. The average hydrodynamic radii of fibrinogen control (0 µM HOCl) and the HOCl-oxidized fibrinogen solution (150 μM HOCl) were 12.3 ± 0.08 nm and 12.2 ± 0.02 nm, respectively (Supplementary Fig. S3b). DLS analysis revealed the homogeneity of fibrinogen solutions after HOCl oxidation and had similar correlation coefficient decay curves (Supplementary Fig. S3c). Regardless of HOCl oxidation, the purified fibrinogen solutions exhibited homogenous, monodisperse scattering and the averaged hydrodynamic radii were not significantly different. These results suggest that the hydrodynamic size of fibrinogen as well as its secondary structure in the prefibrillar state are slightly affected by oxidation and are likely to be poor in predicting fibrillar structural changes.

Fibrinogen diffusion in solution is largely determined by the intrinsic properties of the fibrinogen molecule which remain thermally stable under physiological conditions at room temperature^29^. Since fibrinogen solutions were homogeneous and monodisperse, we measured ^1^H spectra and diffusion coefficient of fibrinogen solutions after exposure to HOCl oxidation by DOSY NMR. Both fibrinogen control and the oxidized fibrinogen solutions showed resonances with a broad outline at amide and aliphatic regions in the ^1^H spectra. The ^1^H signals of fibrinogen solutions were dispersed at the amide region from 6.5 ppm to 8.5 ppm (Fig. S4a) and the fibrinogen methyl protons were observed at the aliphatic region from 0.7 to 0.9 ppm (Fig. S4b). The relative signal amplitude of the fibrinogen methyl protons for the HOCl-oxidized fibrinogen solution (150 µM HOCl) was significantly lower than the fibrinogen control (0 µM HOCl). The methyl protons in the upfield domain region of the spectrum were selected to measure the diffusion coefficient of fibrinogen (Fig. S4b). The bulk water signal was suppressed and thus the diffusion coefficient of bound water in the fibrinogen solutions was measured by DOSY NMR. The diffusion coefficient of fibrinogen control was 2.3 × 10^–11^ m^2^s^−1^ (Fig. S4c), which agreed with the literature value^30^. While the diffusion coefficient of bound water protons (13.4 × 10^–11^ m^2^s^−1^) (Fig. S4c) was one order of magnitude smaller than that of pure water (2.3 × 10^−9^m^2^ s^−1^)^31^. After 150 µM HOCl oxidation, the diffusion coefficients of fibrinogen and water protons were increased to 2.7 × 10^–11^ m^2^s^−1^ and 14.5 × 10^–11^ m^2^s^−1^, respectively. This represents an increase in diffusion mobility for both fibrinogen (17.4% increase) and bound water proton (8.2% increase) (Fig. S4c). Therefore, HOCl oxidation changed the shape of fibrinogen to be more compact and enhanced its diffusion mobility. Numerous studies have demonstrated that oxidative modifications affected the structure of functional sites (αC domains) and conformational rearrangement of fibrinogen^26,32,33^. We previously reported that HOCl oxidation reduced the viscosity of fibrinogen solutions^11,34^. Thus, the diffusion mobility of oxidized fibrinogen was increased as its viscosity was reduced.

### Oxidation alters T_2_ relaxation time of water protons in fibrinogen solution

**Figure 2:**
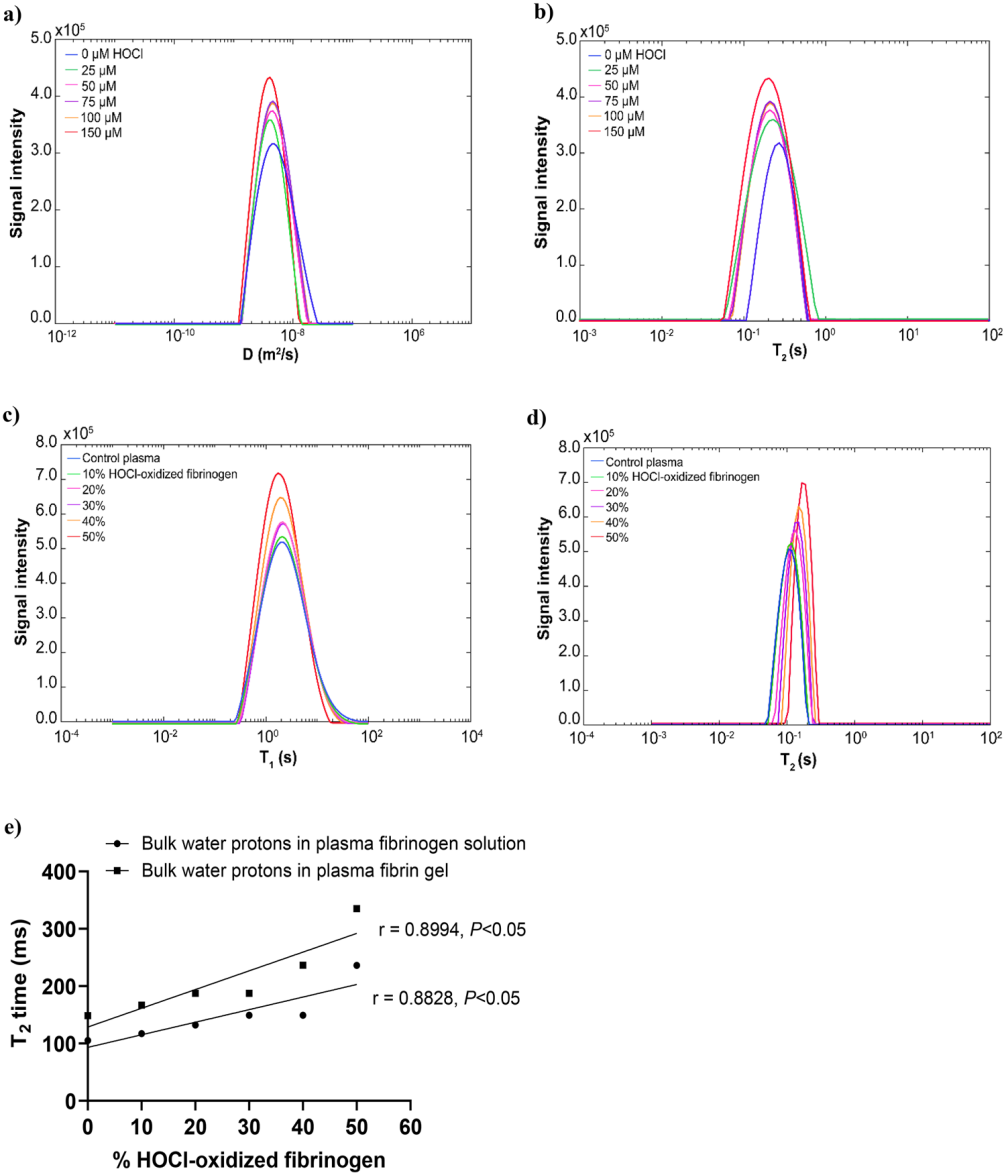
(**a**) Diffusion coefficient (D) distributions of bulk water in the fibrinogen control and the purified fibrinogen solutions oxidized by increasing HOCl concentrations. (**b**) T_2_ distributions of bulk water in the fibrinogen control and the purified fibrinogen solutions oxidized by increasing HOCl concentrations. Diffusion/T_2_ correlations of water protons in the purified fibrinogen solutions oxidized by increasing HOCl concentrations (25, 50, 75, 100, 150 µM). The D/T_2_ correlations were simultaneously measured by PFG for diffusion coefficient (D) and conventional CPMG for T_2_ relaxation time. Fibrinogen solution without oxidation (0 µM HOCl) was used as a fibrinogen control. (**c**) T_1_ distributions of bulk water in the plasma fibrinogen solution and plasma fibrinogen solution added with an increasing percentage of HOCl-oxidized fibrinogen (10, 20, 30, 40, 50%) (pre-oxidation of purified fibrinogen by 150 µM HOCl). (**d**) T_2_ distributions of bulk water in the plasma fibrinogen solution and plasma fibrinogen solution added with an increasing percentage of HOCl-oxidized fibrinogen. Plasma without HOCl-oxidized fibrinogen (0%) was used as control plasma. Plasma fibrinogen solution was indirectly oxidized by adding HOCl-oxidized fibrinogen (10, 20, 30, 40, 50%). The T_1_/T_2_ correlations were simultaneously measured by saturation inversion recovery for T_1_ time and conventional CPMG for T_2_ relaxation time. (**e**) The correlation of bulk water T_2_ relaxation time and the level of indirect oxidation of plasma fibrinogen solution added with an increasing percentage of HOCl-oxidized fibrinogen as well as the correlation of bulk water T_2_ relaxation time and the level of indirect oxidation of plasma fibrin gels formed by increasing percentage of HOCl-oxidized fibrinogen. The correlations of bulk water T_2_ relaxation times and the level of indirect oxidation in plasma fibrinogen solutions or plasma fibrin gels were analyzed by Pearson correlations. *P* < 0.05 is considered as a significant correlation. The 2D-NMR diffusion/T_2_ correlations and T_1_/T_2_ were measured using 600 MHz NMR spectrometry. The multiple D/T_2_ or T_1_/T_2_ water signals were analyzed by 2D inverse Laplace transform algorithm (2DILT) and Iterative Thresholding Algorithm for Multiexponential Decay (ITAMeD).

Two-dimensional D/T_2_ or T_1_/T_2_ were used to determine the correlations between NMR signatures (diffusion coefficient and relaxation times of water signal) and fibers rearrangement in the fibrin clots. Water proton was used as a probe, which allows monitoring of the two-dimensional D/T_2_ correlations signals. The PFG and conventional CPMG with variable 2τ delays were applied for simultaneous measurement of diffusion coefficient (D) and T_2_ relaxation time. The 2D correlational profiles in frequency domains were acquired and encoded sequentially over time. As compared to control, the diffusion coefficient of bulk water protons in the 150 µM HOCl fibrinogen solution was slightly decreased from 4.64 × 10^–9^ m^2^s^−1^ to 4.04 × 10^–9^ m^2^s^1^ (Fig. 2a, Supplementary Table S1) and T_2_ relaxation time of bulk water was decreased from 256.6 ms to 187.4 ms (Fig. 2b, Supplementary Table S1). The fibrinogen was diluted in PBS buffer containing salts (NaCl, KCl), which decreased the water diffusion mobility and increased the T_2_ relaxation rate. It is known that salt ions form a layer surrounding the surface of the particle where the water molecules immobilized, resulting in a faster water T_2_ relaxation rate in saline solution^35^. Bulk water T_2_ becomes shorter when the salt ions interact with water molecules in any way and to any degree^36^. We observed that water molecules bound to the surface of oxidized fibrinogen and resulted in a shorter T_2_ relaxation time.

To examine the similar effects of fibrinogen oxidation taking place in normal pooled human plasma, we determined T_1_/T_2_ correlation profiles of water protons in plasma solutions after indirect oxidation by adding an increasing percentage of HOCl-oxidized fibrinogen (pre-oxidized by 150 µM HOCl). The HOCl-oxidized fibrinogen was added to the plasma fibrinogen solution, which has low salt content, and negligible effects on NMR relaxations^35^. Oxidation did not significantly affect the bulk water T_1_ time (Fig. 2c, Supplementary Table S2) and the diffusion mobility of bulk water in the oxidized plasma fibrinogen solutions was slightly increased by 10% (Supplementary Table S2). Increasing the fraction of oxidized fibrinogen significantly increased bulk water T_2_ relaxation time by twofold from 104.8 ms to 236.5 ms at a threshold of 50% HOCl-oxidized fibrinogen (Fig. 2d, Supplementary Table S2). Bulk water T_2_ relaxation time was positively correlated with the level of indirect oxidation of plasma fibrinogen solution (Fig. 2e). A study has found that the macromolecules in the plasma fibrinogen solution have a different diamagnetic susceptibility than the bulk water^37^. Our results are consistent with previous findings that water diffusion and T_1_ relaxation did not differ significantly between protein solutions and hydrogels^38^.

### Oxidation alters T_2_ relaxation time of water signal in fibrin clots

**Figure 3:**
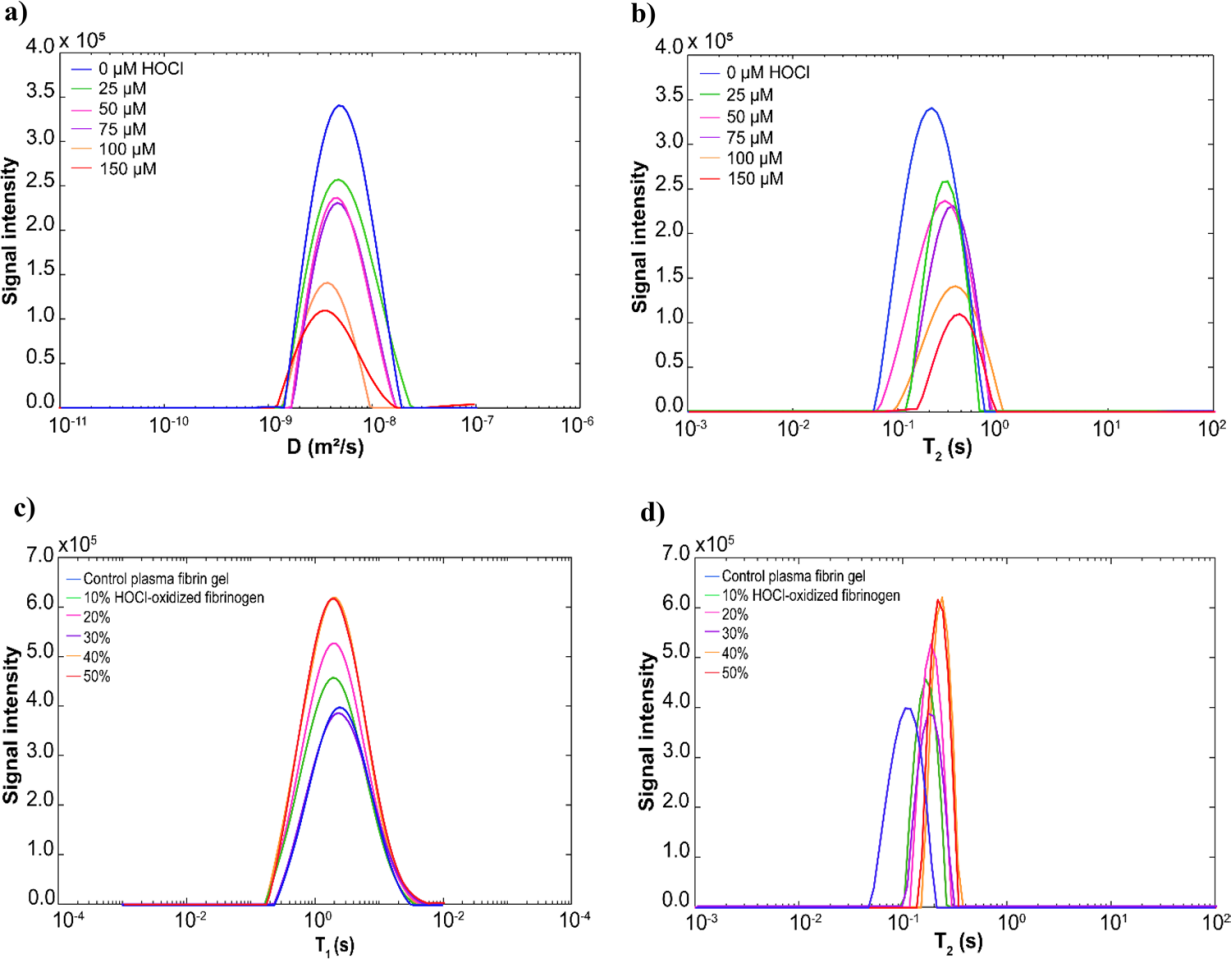
(**a**) Diffusion coefficient (D) distributions of bulk water in the control fibrin gel and fibrin gels oxidized by increasing HOCl concentrations. (**b**) T_2_ distributions of bulk water in the control fibrin gel and fibrin gels oxidized by increasing HOCl concentrations. Diffusion/T_2_ correlations of water protons in the fibrin gels oxidized by increasing HOCl concentrations (25, 50, 75, 100, 150 µM). The D/T_2_ correlations were simultaneously measured by PFG for diffusion coefficient (D) and conventional CPMG for T_2_ relaxation time. Fibrin gel without HOCl oxidation (0 µM HOCl) was used as a control. (**c**) T_1_ distributions of bulk water in the control plasma fibrin gel and plasma fibrin gels formed by adding an increasing percentage of HOCl-oxidized fibrinogen (10, 20, 30, 40, 50%). (**d**) T_2_ distributions of bulk water in the control plasma fibrin gel and plasma fibrin gels formed by adding an increasing percentage of HOCl-oxidized fibrinogen. Plasma fibrin gel without HOCl-oxidized fibrinogen (0%) was used as a control. Plasma fibrin gels were indirectly oxidized by adding HOCl-oxidized fibrinogen (10, 20, 30, 40, 50%) to plasma prior to clotting. The HOCl-oxidized plasma fibrin gels were formed by adding HOCl-oxidized fibrinogen and then incubated with 0.16 NIH U/mL thrombin at 37 °C for 1 h. The T_1_/T_2_ correlations were simultaneously measured by saturation inversion recovery for T_1_ time and conventional CPMG for T_2_ relaxation time.

We used 2D-NMR spectrometry to investigate the effects of HOCl oxidation on the structural rearrangements of fibrin clots. We first examined fibrin clots that were formed from the HOCl-oxidized fibrinogen after activation by thrombin. Compared to control fibrin gel, the diffusion coefficient of bulk water protons in the HOCl-oxidized fibrin gel (150 µM HOCl) decreased from 4.64 × 10^–9^ m^2^s^−1^ to 3.51 × 10^–9^ m^2^s^−1^ (Fig. 3a, Supplementary Table S1). In contrast, the bulk water T_2_ relaxation time in 150 µM HOCl oxidized fibrin gel increased from 211.5 ms to 377.5 ms (Fig. 3b, Supplementary Table S1). The 150 µM HOCl oxidized fibrin gel demonstrated a twofold increase in the T_2_ relaxation time and a 1.3-fold increase in the diffusion coefficient of bulk water. These experiments also showed that 2D-NMR detection threshold is 12% oxidative change in the fibrin structure resulting from 25 µM HOCl (Supplementary Table S1). Water diffusion mobility decreased due to the interaction between water molecules and salts ion present in the oxidized fibrin gel. The T_2_ relaxation time of bulk water was increased as a result of the hydration shell around the fibers compensating for the effects of salt ions on the surface-bound water molecules. When the mobile peptide interacts with the hydrogel matrix, it is slowed down, causing faster peptide proton relaxation^38^. The water T_2_ relaxation in the hydrogel increases linearly with the shear modulus G, whereas the diffusion and T_1_ relaxation are independent of G^38^. We demonstrated that T_2_ relaxation time of bulk water in the control fibrin gel is caused by coupling between bulk water protons and the fibrin hydration shell. We previously reported that HOCl oxidation reduced the shear modulus of fibrin gel^11,34^. Upon oxidation of HOCl, the T_2_ relaxation time of bulk water increased due to a decrease in the stiffness of fibrin gels.

We sought to determine the indirect effect of indirect oxidation of plasma fibrin gels by measuring T_1_/T_2_ correlations of the bulk water protons in the plasma fibrin gels formed by the addition of increased percentages of HOCl-oxidized fibrinogen. In the 50% HOCl-oxidized plasma fibrin gel, the diffusion coefficient of bulk water increased by 10% (Supplementary Table S2), while the T1 relaxation time of bulk water increased 1.3-fold, from 1918 to 2420 ms (Fig. 3c, Supplementary Table S2). The bulk water T_2_ relaxation time in the 50% HOCl-oxidized plasma fibrin gel was shifted twofold from 148.5 ms to 335.3 ms compared to the control (Fig. 3d, Supplementary Table S2). The bulk water T_2_ relaxation time was positively correlated with the level of indirect oxidation of plasma fibrin gels formed by HOCl-oxidized fibrinogen added to plasma fibrinogen solution (Fig. 2e).

**Figure 4:**
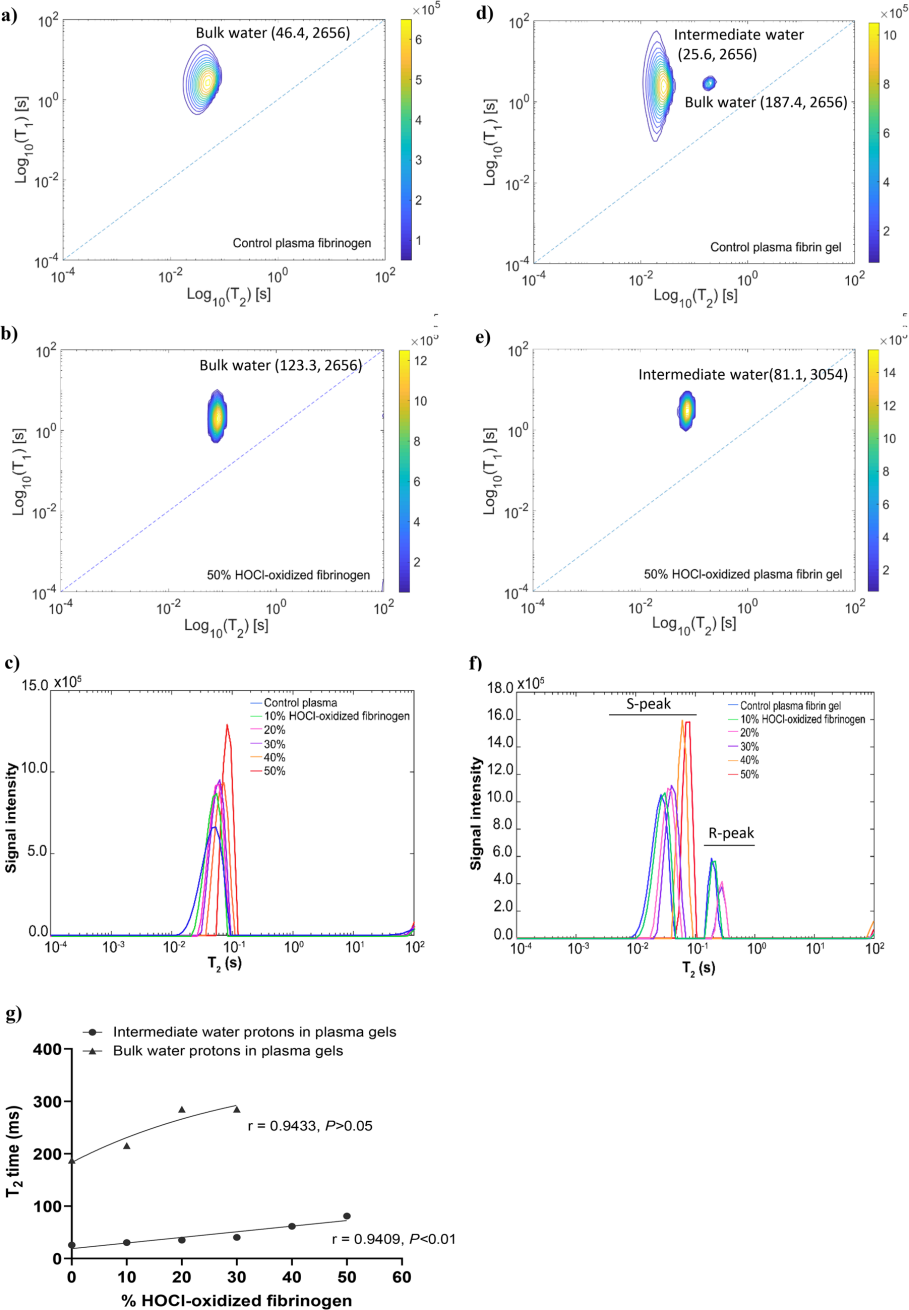
Water protons were measured by a modified CPMG with shorter 2τ delays and saturation inversion recovery pulse sequences for and T_1_/T_2_ correlations spectrum was obtained within few minutes. The fast acquisition of T_1_* /*T_2_ correlations was analyzed by 2D inverse Laplace transform. (**a**) T_2_/T_2_ correlational spectrum of plasma fibrinogen solution control. (**b**) T_2_/T_2_ correlational spectrum of 50% HOCl-oxidized plasma solution. The T_2_/T_2_ correlational spectra of plasma fibrinogen solution control and 50% HOCl-oxidized plasma fibrinogen solutions (added with 50% HOCl-oxidized fibrinogen). The T_1_/T_2_ correlational spectrum was presented in the semilog-log plot. (**c**) T_2_ distributions of bulk water in the plasma fibrinogen solution control and plasma fibrinogen solutions added with an increasing percentage of HOCl-oxidized fibrinogen (10, 20, 30, 40, 50%). Plasma fibrinogen solution without HOCl-oxidized fibrinogen solution (0%) was used as a control. (**d**) T_2_/T_2_ correlational spectrum of control plasma fibrin gel. (**e**) T_2_/T_2_ correlational spectrum of 50% HOCl-oxidized plasma fibrin gel (which formed by adding 50% HOCl-oxidized fibrinogen to plasma fibrinogen solution prior to clotting). The relaxation components in the T_1_/T_2_ correlational spectrum, including the bulk water within the plasma fibrin gel, and intermediate water present in the hydration shell of fibers. (**f**) T_2_ distributions of water protons in the control plasma fibrin gel and plasma fibrin gels formed by adding an increasing percentage of HOCl-oxidized fibrinogen (10, 20, 30, 40, 50%). Plasma fibrin gel without HOCl-oxidized fibrinogen solution (0%) was used as a control. (**g**) The correlations between bulk water T_2_ relaxation times and the level of indirect oxidation of plasma fibrin gels formed by adding an increasing percentage of HOCl-oxidized fibrinogen (10, 20, 30, 40, 50%) as well as the correlations between intermediate water T_2i_ relaxation times and the level of indirect oxidation of plasma fibrin gels formed by adding an increasing percentage of HOCl-oxidized fibrinogen (10, 20, 30, 40, 50%). The correlations of water T_2_ relaxation times and the level of indirect oxidation in plasma fibrin gels were analyzed by Pearson correlations. *P* < 0.05 is considered as a significant correlation.

Next, we used a 2D-NMR sparse sampling method (2D-NMR with fast acquisition) which provides similar results within a shorter time frame than conventional T_1_/T_2_ correlations experiments. A recent study reported that water peaks (A-ratio) in the T_1_/T_2_ correlation spectra can be used to predict the degree of water-protein interactions in the fibrinogen-plasma system^17^. Compared to plasma fibrinogen control (Fig. 4a), the 50% HOCl-oxidized plasma fibrinogen solution exhibited a bulk water peak with higher intensity (Fig. 4b). The T_2_ distributions revealed that the bulk water T_2_ relaxation time in the 50% HOCl-oxidized plasma fibrinogen solution increased by twofold, from 46.4 ms to 123.3 ms, compared to control (Fig. 4c, Supplementary Table S3). The control plasma fibrin gel consisted of bulk water (T_2_) within the fibrin gel (Fig. 4d), and intermediate water molecules (T_2i_) present in the hydration shell of fibers. It is known that bulk water is an exchangeable peak^39^ and makes a major contribution to the T_2_ relaxation^40^. After oxidation, the bulk water was disappeared, and the intermediate water peak became more intense (Fig. 4e), suggesting that T_2_ relaxation of the oxidized plasma fibrin gel was dominated by the water fraction in the hydration shell of fibers. The intermediate water T_2i_ time in the 50% HOCl-oxidized plasma fibrin gel was increased by threefold, from 25.6 ms to 81.1 ms, compared to control (Fig. 4f, Supplementary Table S3). The T_2_ relaxation time of intermediate water (T_2i_) was positively correlated with the level of indirect oxidation of plasma fibrin gel formed by adding an increasing percentage of HOCl-oxidized fibrinogen. While the T_2_ relaxation time of bulk water (T_2_) increased with lower indirect oxidation of plasma fibrin gel (added with 30% HOCl-oxidized fibrinogen or less) (Fig. 4g). Gelation induces faster relaxation of T_2_ by generating local magnetic field inhomogeneity, resulting from fiber formation and growth^38^. The T_2_ relaxation rate is significantly influenced by local magnetic field inhomogeneity, but T_1_ remains unchanged^41^***.*** Our results showed that oxidation increased water T_2_ relaxation time, which is attributed to a higher fraction of water in the hydration shell. The oxidized plasma fibrin gels are packed with thinner fibers, causing structural heterogeneity and reducing the gels' stiffness. The thinner fibers do not release water from the hydration shell into the bulk, increasing T_2_ relaxation time.

**Figure 5:**
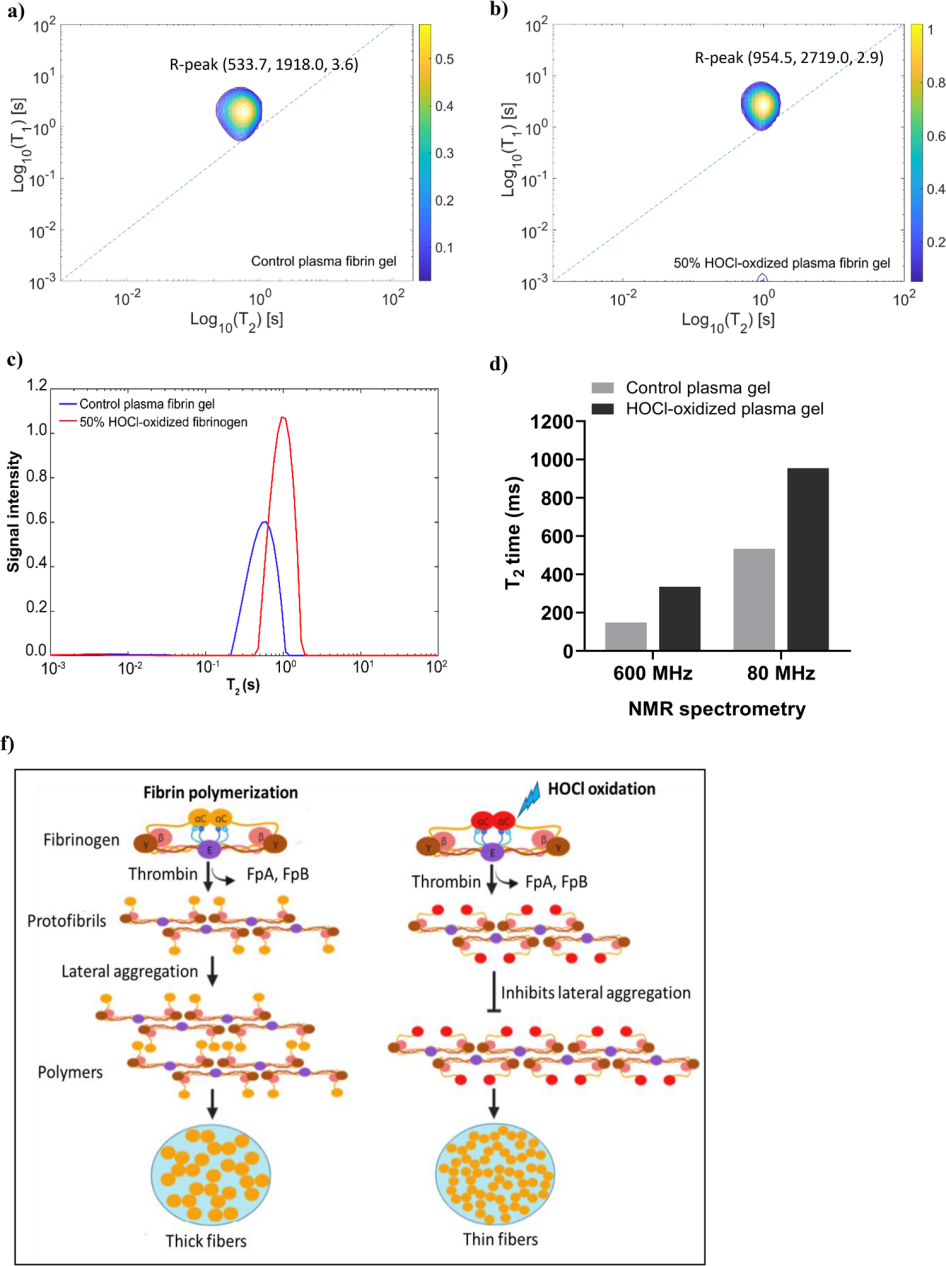
The T_2_/T_2_ correlational spectra of control plasma fibrin gel and 50% HOCl-oxidized plasma fibrin gel measured by Bruker mq20 Minispec benchtop spectrometry operating at 80 MHz. (**a**) T_2_/T_2_ correlational spectrum of control plasma fibrin gel. (**b**) T_2_/T_2_ correlational spectrum of 50% HOCl-oxidized plasma fibrin gel (formed by added with 50% HOCl-oxidized fibrinogen prior to clotting). The T_1_/T_2_ correlational spectrum was presented in the semilog-log plot. (**c**) T_2_ distributions of water in the control plasma fibrin gel and 50% HOCl-oxidized plasma fibrin gel. (**d**) T_2_ relaxation times of water protons in the control plasma fibrin gel and 50% HOCl-oxidized plasma fibrin gel were measured by both 80 MHz and 600 MHz NMR spectrometry. The 80 MHz spectrometry acquired eight scans for the signal averaging in each experiment, 36 data points collection time of 1 min echo time, and 6000 number of echoes. The 600 MHz spectrometer used a conventional CPMG and saturation inversion recovery pulse sequences. (**f**) The diagram illustrates the proposed mechanism of protofibrils lateral aggregation to form fibers in the plasma fibrin gels upon HOCl oxidation.

Lastly, we tested whether low field NMR spectrometry (a benchtop NMR operating at 80 MHz) can identify changes in the gel structure due to oxidation. Using this method, the bulk water peak in the 50% HOCl-oxidized plasma gel was shifted to a longer T_2_ relaxation time (Fig. 5b) compared to the control plasma fibrin gel (Fig. 5a,b). The T_1_/T_2_ correlation distributions showed that bulk water T_2_ relaxation time increased by twofold from 533.7 ms to 954.5 (Fig. 5c). The bulk water T_1_ relaxation time in oxidized plasma fibrin gel was slightly longer. The results indicate that low field NMR spectrometry can be used to distinguish oxidized fibrin gel from control, and its sensitivity is comparable to high field NMR spectrometry (Fig. 5d).

## Discussion

Our results show that (i) T_2_ relaxation of water protons can probe the structural alteration of plasma fibrin clots due to the oxidation at the level of at least 12% or higher. (ii) T_2_ relaxation of water (and not T_1_ or diffusion coefficient) can be used as the most sensitive readout that correlates with oxidation. (iii) Detection can be achieved using high field NMR spectrometers as well as benchtop NMR systems operating at least 80 MHz. Measuring T_2_ of water in the fibrin gel is typically fast and can be performed in situ within several minutes as opposed T_1_ and diffusion requiring longer acquisition times. It was reported that the total oxidation level in the plasma from patients with trauma coagulopathy was about 9% when focusing solely on methionine oxidation of soluble prefibrillar fibrinogen (*γ*M78, B*β*M367, and A*α*M476)^11,21^. On the wound site, neutrophils are recruited and a fibrin gel with a higher level of oxidation is deposited. Since fibrin gel concentrates within clots, our NMR method is still able to detect the changes in T_2_ relaxation time from oxidation-induced structural changes when examining clotted fibrin samples, thus overcoming the limitations of plasma fibrinogen samples. Therefore, plasma fibrin gel is preferable for 2D-NMR analysis.

Our results provide novel experimental evidence for the impact of oxidation on fibrinogen polymerization. These data support that HOCl oxidation alters fibrinogen polymerization similar to the inhibition of AαC domain interactions during fibrin clot formation. Fibrinogen with truncated αC regions (Aα251) forms fibrin clots composed of thinner fibers, decreased stiffness, and enhanced fibrinolysis^12^. A recombinant hybrid fibrinogen variant in which the human αC regions were substituted with homologous chicken αC sequences that lacking the ability to laterally aggregate beyond single-stranded protofibrils^13^. Competitive inhibition of lateral aggregation was also found in the presence of αC fragments^42^. We used a recombinant human αC fragment (AαC 419–502) as a titratable model of the fibrin polymerization that takes place without αC domain interactions. In each case, the indicators were thin fibered clots with altered stiffness and susceptibility to fibrinolysis. Our results showed that the effect on clot turbidity was nearly identical to that seen with oxidation. This observation supports MD simulations pointing towards a potential mechanism by which oxidation of the AαC domain inhibits lateral protofibril aggregation due to the partial burial of local polymerization binding sites^26^, making the lateral aggregation of fibrin monomers energetically unfavorable^32,43^.

Water protons are easily be measured using 2D-NMR to detect molecular and morphological changes in aggregates via proton relaxation times^44^. The T_2_ relaxation time is determined by water-water interactions and correlates with the morphological changes or aggregate assemblies^18,45^. Increased water-protein interactions drastically reduce the motion of the water proton, leading to reduced relaxation times for T_1_ and T_2_. It appears that T_2_ relaxation is more effective than T_1_ relaxation^17^. Fibers are formed when fibrinogen monomers aggregate into protofibrils, resulting in structural heterogeneity within the fibrin gel^38,41^. The fibrinogen motions are slowed down, leading to faster relaxation of fibrinogen protons^38^. The coupling between bulk water proton and fibrinogen proton also affects water proton relaxation^38^. Our polymerization experiments showed that HOCl oxidation hindered protofibril lateral aggregation and produced thinner fibers (Fig. 1a,e). The T_1_ relaxation time and diffusion coefficient of bulk water in the plasma fibrinogen solution did not change significantly as the correlation time determining the fibrinogen motion. Fibrinogen solution and fibrin gel appeared to have a relatively small discrepancy in T_1_ relaxation time^36^. The water diffusion and T_1_ relaxation of the hydrogel are independent of the shear modulus G, while the T_2_ relaxation increases linearly with G^38^. We demonstrated that the bulk water T_2_ relaxation time was significantly increased in the oxidized plasma fibrin gels, albeit with a small increase in T_1_ and water diffusion mobility. The bulk T_2_ relaxation time correlated positively with the indirect oxidation of plasma fibrin gel (Fig. 2e). The water in the fibers' hydration shell dominated the T_2_ relaxation of oxidized plasma fibrin gel. Consequently, T_2_ relaxation time of intermediate water (T_2i_) is increasing, while the bulk water T_2_ time is decreasing. The thinner fibers in the oxidized fibrin gel may release fewer water molecules into the bulk, increasing the T_2_ time. The water molecules in the hydration shell contribute to the stability of fibrin clots and create additional water bridges between the fibers^46^. It has been proposed that immobilized water in the hydration layer is linearly proportional to polymers^35^. Hence, the high polymer density could account for the decreased water T_2_ relaxation^35^. Our previous studies found that HOCl oxidation reduces the shear modulus of fibrin gel^11,34^. Therefore, HOCl oxidation reduces the stiffness of fibrin gel leading to an increase in bulk water T_2_ time.

We previously reported that oxidation of AαM476 shifted the equilibrium between the open and closed conformations of the αC domains^26^. The AαM476 residue is essential for dimerization of αC domains and that its oxidation renders the dimerization energetically unfavorable compared to the native structure^43^. We postulate that the largest bulk water T_2_ time is associated with the self-assembly of thinner fibers; while the self-assembly of thick fibers has a shorter T_2_ time and will dominate the water proton relaxation. The protofibril aggregates form open polymers that grow and form thick fibers; however, HOCl oxidation produces closed polymers that inhibit lateral aggregation, resulting in thinner fibers (Fig. 5f).

We propose that T_2_ relaxation of water signal can serve as an NMR signature to capture the arrangement of the fibers and structural properties of the fibrin gels. We anticipate that NMR spectrometry can be a potential device to rapidly identify the contribution of oxidation to coagulopathy after trauma.

## Methods

### Sample preparation

Human fibrinogen (plasminogen and von Willebrand depleted, 99% clottability) and human-α-thrombin were purchased from Enzyme Research Laboratories (#FIB 2). Human plasminogen (#16–16-161200, Athen Research and Technologies) and tranexamic acid (TXA) (Precision Technologies) were purchased. We purchase the commercially available recovered plasma frozen within 8 h (#PS100, Seraplex Inc). Lyophilized human fibrinogen was dissolved in phosphate buffer saline (PBS; 137 M NaCl, 2.7 mM KCl, 8 mM Na_2_HPO_4_12H_2_O, 1.5 mM KH_2_PO_4_, and pH 7.4). Its concentration was determined spectrometrically at 278 nm using an extinction coefficient of 15.1 for 10 mg/mL solution^47^. Hypochlorous acid (HOCl) refers to a mixture of HOCl and OCl^-^ species. The hypochlorite concentration was determined spectrophotometrically using a wavelength of 292 nm (ε = 350 M^−1^ cm^−1^)^48^. All chemicals were of reagent grade and were purchased from Sigma-Aldrich, unless specified otherwise.

### Fibrinogen oxidation

The fibrinogen was incubated with different concentrations of HOCl (0, 10, 25, 50, 75, 100, 125, 150 μmol/L) for 1 h at 37 °C. Fibrinogen was oxidized by HOCl at μmol/g of protein. The oxidation reaction was quenched with a 10 times molar excess of L-methionine. The non-oxidized fibrinogen (0 µmol HOCl) was used as a control. The control sample was made to have the same composition as the 150 μmol/L oxidized sample by addition of methionine. After oxidation, the samples were separated into aliquots and stored at − 80 °C. It should be noted that the addition of methionine and HOCl, which were required for the oxidation reaction, caused a negligibly small increase (< 3%) in the ionic strength of the protein solutions. However, this small increase is not expected to significantly affect the fibrin gel morphology^49^. Fibrinogen concentration at 2 mg/mL in PBS, pH 7.4 was used in all the experiments, unless specified otherwise.

### Spectrophotometric measurements (turbidity assay)

Fibrin polymerization was measured by monitoring turbidity changes with time at 350 nm at 37 °C for 1 h using a UV–Vis spectrophotometer (Cytation 5 Cell Imaging Reader, BioTek). Fibrin polymerization was initiated by the addition of 100 μL of 2 × coating buffer (0.14 M NaCl, 2 mM CaCl_2,_ and 44 mM Hepes buffer at pH 7.4) containing 0.16 NIH U/mL thrombin into 100 μL of 2 mg/mL oxidized or fibrinogen control solution. PBS was used as the blank. The turbidity changes of the samples in the process of gelation were kinetically measured for 1 h. The turbidity curves were characterized using the following parameters: (1) the lag time, measured as the time elapsed until an increase in absorbance was seen, which reflects the time to the start of lateral fibril aggregation after cleavage of fibrinopeptides by thrombin; (2) the maximal slope (Vmax), calculated as the slope of the steepest part of the polymerization curve, which represents the rate of lateral protofibril association the rate of protofibril aggregation into fibers; (3) the maximal turbidity of the growing clot, recorded 60 min after polymerization was initiated, which reflects fibrin fiber diameter and the number of protofibrils per fiber (turbidity was correlated with the thickness of an individual fiber)^22^.

### Thrombin and reptilase time assays

The STart Max coagulation analyzer (Diagnostica Stago) was used to measure the time required for a fibrin clot to form following the addition of a standard amount of thrombin and/or reptilase. Thrombin time measures the clotting time required for a clot to form at 37 °C after the addition of thrombin which activates the conversion of fibrinogen to fibrin by cleavage of fibrinopeptides A and B. Reptilase time uses batroxobin, a viper venom, to measure the clotting time required for fibrinogen converted to fibrin by cleavage of fibrinopeptide A. The normal value of thrombin time and reptilase time are < 21 s and < 24 s, respectively.

### Clauss fibrinogen assay (clottability)

The Clauss fibrinogen assay is used to measure the concentration of functional fibrinogen. It is performed on a dilution of fibrinogen samples to eliminate interference by substances such as heparin and fibrin degradation products. The sample was diluted at 1:20 with Owrens–Kohler. Fibrinogen concentration was quantified by the addition of STA-Liquid Fibrinogen reagent that containing human thrombin of 100 IU/mL to the diluted sample. The diluted fibrinogen is clotted with high concentration thrombin, the resulting time to clot formation, typically measured either mechanically or optically, is directly proportional to the concentration of clottable fibrinogen in the sample. A standard curve of known fibrinogen concentration vs. clotting time is then used to determine the concentration of fibrinogen, of which fully functional fibrinogen is typically over 95% clottable. The STart Max coagulation analyzer (Diagnostica Stago) automatically converted the clotting time measured in seconds to fibrinogen concentration in mg/dL using a [log–log] fibrinogen standard curve. The normal fibrinogen level is at the range of 2–4 mg/mL (200–400 mg/dL).

### SDS-PAGE

SDS-PAGE was performed according to Laemmli^50^. The purified fibrinogen solution was oxidized by increasing HOCl concentrations (0, 10, 25, 50, 75, 100, 125, 150 µM), and the electrophoretic patterns of the fibrinogen protein after HOCl oxidation were analyzed by SDS-PAGE. The reduced samples were prepared by adding 4 × Laemmli sample buffer (Bio-Rad) containing 5% 2-mercaptoethanol and heated at 95 °C for 10 min. 5 µg of protein sample and 5 µL of protein marker (Precision Plus Protein™ Dual Color Standards) was loaded into the respective lane. Protein was separated on a 12% SDS-PAGE for 2 h at a constant voltage of 100 V using Mini-PROTEAN Tetra Cell (Bio-Rad). The gel was stained with Coomassie brilliant blue R-250 for 4 h and destained in distilled water for 2 h. Non-oxidized fibrinogen was used as a control.

### Circular dichroism (CD)

Fibrinogen control (0 µmol/L HOCl) and the oxidized fibrinogen samples (10, 25, 50, 75, 100, 125, 150 μmol/L) were prepared at a concentration of 0.025 mg/mL in 50 mM sodium phosphate buffer (pH 7.2) and transferred to the 1 mm pathlength quartz cuvette for CD spectra measurement (Chirascan V100). CD spectra of fibrinogen control and the oxidized fibrinogen samples were recorded over wavelengths spanning between 280 and 200 nm at 25 °C were recorded. Triplicates were done with 0.5 μs time per incremental scan. Analysis of the CD spectra was performed using the secondary structure prediction program supplied with the spectropolarimeter. Molar ellipticity values [q] were calculated according to the equation: [θ] (deg-cm^2^ dmol^−1^) = [θ (MRW)]/[10(l)(c)], where θ is the displacement from the baseline value X to the full range in degrees; MRW is the mean residue weight of the amino acids; (l) is the path length of the cell (cm); and (c) is protein concentration (g/mL). All CD data were expressed as the mean residue ellipticity [θ], in units of degrees square centimeter per decimole.

### Dynamic light scattering (DLS)

DLS analysis was performed at 25 °C using a Malvern Zetasizer Nano series instrument. DLS was used to determine the effect of HOCl oxidation on the hydrodynamic radius and size distribution of fibrinogen at physiological conditions i.e., 2 mg/mL at pH 7.4 and ionic strength of 0.15 M NaCl. The aggregation of fibrinogen with increasing concentration (2–10 mg/mL) was also determined.

### Protein expression and purification of recombinant human AαC domain

A recombinant αC fragment corresponding to human fibrinogen αC-domain (AαC 419–502 residues) was produced in *E. coli* using pET-28b expression vector as described earlier^51^. The pET-28b vector carrying the coding sequences of His-tagged AαC 419–502 fragment was synthesized (Bio Basic Asia Pacific). It consisted of 6 × His-linker-TEV cleavage (MGHHHHHHMGNSENLYFQ) and the coding sequences (GDKELRTGKEKVTSGSTTTTRRSCSKTVTKTVIGPDGHKEVTKEVVTSEDGSDCPEAMDLGTLSGIGTLDGFRHRHPDEAAFFDT). The plasmid was transformed into Rosetta 2(DE3) Singles *E. coli* host cells (Novagen). The cDNA fragment was sequenced in both directions to confirm the integrity of the coding sequences. Cells were grown in terrific broth at 37 °C for 3–4 h until OD_600_ reached 0.8–1.0 and followed by induction with 0.5 mM Isopropyl β-D-1-thiogalactopyranoside (IPTG). The cells were induced and propagated at 18 °C overnight. The cell pellet was harvested and dissolved in TBS (20 mM Tris–HCl buffer containing 150 mM NaCl and 0.1 mM PMSF, pH 8.0). The protein was then lysed by a sonicator for several pulse cycles. The His-tagged Aα419–502 fragment was prepared from the soluble fraction of the bacterial lysate^51^. The soluble His-tagged protein was incubated with Ni–NTA resin at 4 °C overnight and purified through immobilized metal affinity chromatography (IMAC). The His-tagged Aα419–502 protein was eluted and concentrated using an Amicon ultra-15 centrifugal filter (molecular mass cutoff of 3 kDa) and desalted through a PD-10 column (GE Healthcare) equilibrated with TBS at pH 7.4. The recombinant protein was purified and fractionated by fast performance liquid chromatography (FPLC) (ÄKTA pure, GE Healthcare) on a Superdex75 10/300 GL column equilibrated with TBS at pH 7.4. The purified AαC 419–502 protein was concentrated to 10–15 mg/mL using an Amicon ultra-15 centrifugal filter (Millipore) and stored at -80 °C prior analysis. Purity of native AαC 419–502 fragment (without reduction by 2-mercaptoethanol) was analyzed by 15% SDS-PAGE.

### Protein concentration determination

The concentration of the recombinant human AαC 419–502 fragment was determined spectrophotometrically at 280 nm using extinction coefficient E^1%^ = 1.27 calculated from the amino acid composition with the equation: E^1%^ = (5,690 W + 1,280Y + 120S-S)/(0.1 M), where W, Y, and S–S represent the number of Trp and Tyr residues and disulfide bonds, respectively, and M represents the molecular mass^52^. The amino acid sequence of the recombinant human AαC 419–502 fragment was analyzed by ProParam (Swiss-Prot).

### Mass spectrometry analysis

The intact AαC 419–502 fragment was prepared, and its molecular mass was determined by ESI-TOF mass spectrometer for molecular mass determination of proteins (Agilent Technologies). The samples were analyzed by the Proteomic Core Facility of the Biological Research Center (BRC). The data was acquired at the rate of 1 spectrum/sec and the acquisition window was set from *m/z* 100 to 3000. The peaks in the total ion chromatogram (TIC) were integrated and the mass spectra at 180 fragmentor voltages were obtained. The multiple charge state distributions of the intact proteins were deconvoluted using the Maximum Entropy deconvolution algorithm.

### Diffusion coefficient of water and fibrinogen protons measured by DOSY NMR

The ^1^H DOSY (diffusion-ordered spectroscopy) experiments were carried out at 25 °C on a Bruker DRX 600-MHz spectrometer equipped with a cryoprobe and a standard *z*-gradient inverse probe head (TXI, 5 mm tube) capable of producing gradients with a maximum strength of 53 G/cm. ^1^H DOSY is composed of a stimulated-echo sequence incorporating bipolar gradient pulses and a longitudinal eddy current delay (PFG)^53^. Water signal was suppressed by WATERGATE pulse sequence (water suppression by gradient-tailored excitation) and the diffusion coefficient of bound water and fibrinogen protons were analyzed. The amplitude of field gradient was varied from 2 to 95% of G_max_ over 32 steps increment under constant diffusion time (50 ms). A gradient recovery delay of 0.2 ms and an eddy current delay of 5 ms were used. The samples for DOSY experiment were prepared by addition of 10% D_2_O (v/v) and 0.5 mM 4, 4- dimethyl-4-silapentane-1-sulfonic acid (DSS) into the purified fibrinogen solution oxidized by an increasing concentration of HOCl oxidation. The native fibrinogen solution without HOCl oxidation was used as a control. The chemical shift region 0.7–0.9 ppm that encompassed strong signals for fibrinogen methyl protons was chosen for measurements. This region was selected to eliminate potential errors in the peak integrals arising from disturbances of the water signal and to avoid extra complications from overlapping signals. The diffusion coefficient of the bound water signal in the fibrinogen solution was also measured. The chemical shifts of the ^1^H resonances were referenced to the DSS signal. The ^1^H DOSY acquired data was collected and processed using Bruker Topspin 3.5 software. The diffusion coefficient of the samples were analyzed by NMRgenerator^54^ and computer-aided resonance assignment (CARA)^55^.

### Diffusion/T_2_ and T_1_/T_2_ relaxation times of water signals measured by 2D-NMR

Plasma fibrinogen solution was added with an increasing percentage of HOCl-oxidized fibrinogen (pre-oxidized purified fibrinogen by 150 µM HOCl) and supplemented with 10% D_2_O. Plasma fibrinogen solution without HOCl-oxidized fibrinogen was used as control. Samples were gently mixed and transferred into 3 mm NMR tubes. Plasma fibrin gels were prepared by addition of 1.0 μL of 0.2 mol/L CaCl_2_ solution, 1.0 μL of thrombin (final concentration 0.4 NIH U/mL), and 10% D_2_O to 200 μL of HOCl-oxidized plasma fibrinogen solution. Gelation of the sample took place inside a 3 mm NMR tube after mixing. HOCl-oxidized plasma fibrin gels are composed of an increasing percentage of HOCl-oxidized fibrinogen. Plasma fibrin gel without HOCl-oxidized fibrinogen was used as a control. The plasma fibrinogen solutions and fibrin gels were used for 2D-NMR measurements of the ^1^H diffusion/transverse relaxation (D/T_2_) and longitudinal/transverse (T_1_/T_2_) relaxation correlations. High filed 2D-NMR were performed at 25 °C on a Bruker Avance III 600 MHz spectrometry. Diffusion coefficient data were acquired using PFG with encoding delay of 1 ms, diffusion delay of 50 ms, and maximum gradient strength of 53 G/cm. T_1_/T_2_ correlation data were acquired using a Carr-Purcell-Meiboom-Gill (CPMG)^19^ and saturation inversion recovery pulse sequences with an echo time of 2·τ_2_ = 1.92 ms, and inversion time τ_1_ logarithmically spaced from 1 ms to 50 s. The number of echo for T_1_ and T_2_ were 32 and 38, respectively. The accumulated signals were collected from 1 scan of the data points in the multi-dimensional spectra. Firstly, the 2D-NMR data were acquired using a conventional CPMG with variable 2τ delays and saturation recovery pulse trains. Next, the multi-dimensional T_1_/T_2_ correlation was acquired based on sparse sampling of time dimensions^56,57^ using a modified CPMG with shorter 2τ delays and saturation inversion recovery pulse sequences. Echo signals were acquired between two adjacent 180° pulses. This sparse sampling 2D-NMR method allows efficient reconstruction of a 2D spectrum in an experimental time frame and enables fast acquisition of 2D-NMR relaxation data for 2D inverse Laplace transform. Whereas the conventional CPMG decay curves and the saturation inversion recovery were analyzed by 2D inverse Laplace transform algorithm (2DILT) and Iterative Thresholding Algorithm for Multiexponential Decay (ITAMeD) to deconvolute multiple D/T_2_ or T_1_/T_2_ signals as previously described^56,57^. These algorithms exploit the principle of compressed sensing for sparse sampling, processing, resolving, and reconstruction of the D/T_2_ and T_1_/T_2_ correlations. The accumulated signal intensity data were converted to logarithms. CONTIN-generated diffusion, T_1,_ and T_2_ distributions were obtained from the continuous ILT of the multi-exponential decay curves. The number of exponential decays was fixed to three for all samples. Water proton was measured without water suppression. Water T_2 _was the dominant component, accounting for > 90% of the total CPMG signal intensity^19^. D/T_2_ and T_1_/T_2_ correlations were analyzed by MATLAB software.

### Low field 2D-NMR measurement of water protons in plasma fibrin gel

The T_1_/T_2_ relaxation times of water protons in the control plasma fibrin gel and plasma fibrin gel added with 50% HOCl-oxidized fibrinogen were measured at 25 °C using a Bruker mq20 Minispec benchtop spectrometry operating at 80 MHz. Plasma fibrin gels were prepared by addition of 2.0 μL of 0.2 mol/L CaCl_2_ solution, 2.0 μL of thrombin (final concentration 0.4 NIH U/mL), and 10% D_2_O to 500 μL of plasma fibrinogen solution added with 50% HOCl-oxidized fibrinogen. Plasma fibrin gel without HOCl-oxidized fibrinogen was used as a control. Gelation of samples took place inside the 5 mm NMR tube after mixing. Samples were loaded into a 5 mm NMR tube and inserted into a 10 mm NMR outer tube for measurement of ^1^H water T_1_/T_2_ relaxation times. A modified saturation inversion recovery and CPMG^19^ pulse sequences were used for the measurement of longitudinal/transverse (T_1_/T_2_) relaxation correlations. Eight scans were used for the signal averaging in each experiment, and 36 data points collection time of 1 min echo time and 6000 number of echoes.

### Electrostatic potential calculations

The electrostatic potential of AαC 419–502 at experimental condition (150 mM NaCl ionic strength at pH 7.4, 25 °C) were calculated by adaptive Poisson–Boltzmann solver (APBS) plugin in PyMOL^58^. The input PQR files were generated by PDB2PQR server^58^. The grid dimensions were automatically set by the APBS plugin according to the dimensions of input AαC 419–502 structure. The electrostatic potentials were calculated by solving the nonlinear Poisson-Boltzmann equation with a single Debye and Hückel (DH) sphere boundary condition. The solvent accessible surface area was calculated using a solvent radius of 1.4 Å. The alignment of amino acid residues of AαC 419–502 was analyzed by BLAST and PASTA 2.0^59^. The secondary structure model of AαC 419–502 was predicted by I-TASSER server^27^.

### Statistical analysis

All experiments were performed in triplicate from three independent experiments unless otherwise stated. Data were expressed as mean ± standard deviation. The comparisons between HOCl oxidation of plasma fibrinogen solutions or plasma fibrin gels and their respective controls were assessed using the ANOVA-Bonferroni test. The statistically significant difference vs control was at the *P* < 0.05 level. The correlation between the T_2_ relaxation time and indirect oxidation levels of plasma fibrinogen solutions or plasma fibrin gel (by addition of HOCl-oxidized fibrinogen) was analyzed by Pearson correlation. All the data were analyzed using Excel and GraphPad Prism.

## Supplementary Information

Supplementary Information.

## Data Availability

The datasets generated and/or analyzed during the current study are available from the corresponding author on reasonable request.
