# Supplementary material for: Tracking oxidation-induced alterations in fibrin clot formation by NMR-based methods
Source: Sci Rep. 2021 Aug 3;11:15691. doi: 10.1038/s41598-021-94401-3 (PMC8333047; doi:10.1038/s41598-021-94401-3)
Supplement: Supplementary file 1 — Supplementary Information. [file 41598_2021_94401_MOESM1_ESM.pdf]

Supplementary Information

Tracking oxidation-induced alterations in fibrin clot formation by NMR-based methods

Wai-Hoe Lau[1], Nathan J. White[2], Tsin-Wen Yeo[1,4], Russell L. Gruen[3*], Konstantin Pervushin[5*]

[1]Lee Kong Chian School of Medicine, Nanyang Technological University, Singapore

[2]Department of Emergency Medicine, University of Washington School of Medicine, Seattle, Washington

[3]ANU College of Health and Medicine, The Australian National University, Canberra ACT, Australia

[4]Communicable Diseases Centre, Institute of Infectious Disease and Epidemiology, Tan Tock Seng Hospital, Singapore

[5]School of Biological Sciences, Nanyang Technological University, Singapore

*Corresponding authors. E-mail address: russell.gruen@anu.edu.au, kpervushin@ntu.edu.sg

Supplementary Figure S1

a)

```
1         10        20        30        40          51       60        70        80
GDKELRTGKEKVTSGSTTTTRRSCSKTVTKTVIGPDGHKEVTKEVVTSEDGSDCPEAMDLGTLSGIGTLDGFRHRHPDEAAFFDT
DDDDDDDDDDDDDDDDDDDDDDDOOOOOOOOOOOOOOOOOOOOOOOOOOOOOOOOOOOOOOOOOOOOOOOOOOOOOOOOOOOOOOOOOODDD
CCCBBBCCCBBBBCCCCBBBBBCCCCBBBBBBBCCCCCBBBBBBBBBCCCCCCCCHHCCCCCCCCCBCCCCCCCCCCCCCBBBCC
-------------------------------------------------------------------------------------
```

```
-: Non aggregating residue
D: Disordered residue
O: Ordered residue
H: α-Helix residue
B: β-strand residue
C: Coiled residue
```

b) **c)**

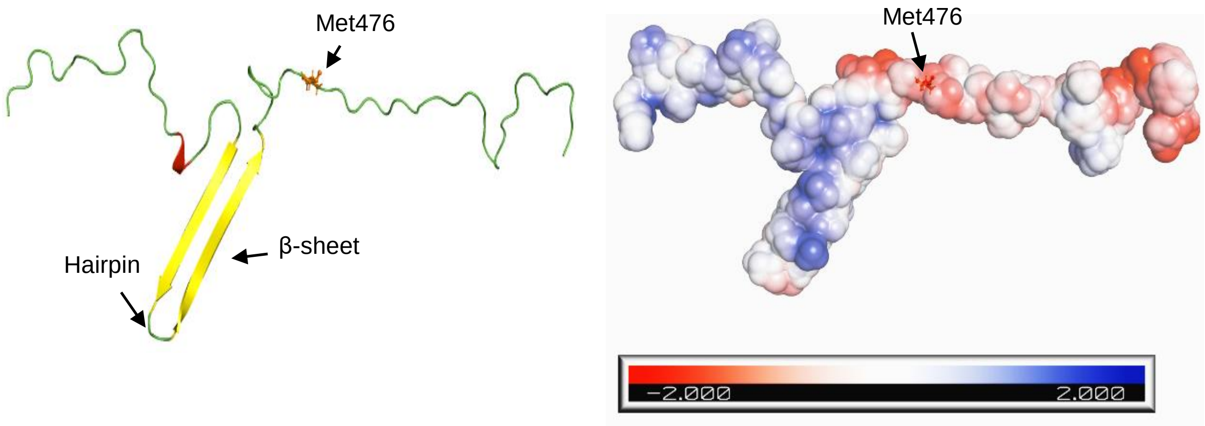

Figure S1. **a**) The amino acid sequence of recombinant human fibrinogen AαC419 - 502 (AαC fragment) composed of α-helix residues (H), β-strand residues (B), and coiled residues (C). It contained disorder (D) structure at the C-terminal and ordered (O) structure at the N-terminal. The amino acid residues sequence of AαC fragment was analyzed by PASTA 2.0 server (http://old.protein.bio.unipd.it/pasta2/index.html). The AαC fragment consisted of 6x His-linker-TEV cleavage (MGHHHHHHMGNSENLYFQ) and followed by the coding sequences. **b**) Homolog model of human AαC fragment was predicted by I-TASSER server (https://zhanglab.dcmb.med.umich.edu/I-TASSER/) and analyzed by PyMOL. AαC fragment exhibited a αC-domain consisting of β-sheet, hairpin, and a Met476 residue at the N-terminal sub-domain. The side chain of Met476 is shown as stick and ball representation. **c**) Electrostatic potential surface of AαC fragment was analyzed by APBS plugins in PyMOL. Electrostatic potential surface of AαC fragment showed a local concentration of negative charge at the N-terminal sub-domain and Met476 residue was exposed on the surface. Negative charge indicated in red, positive in blue, and neutral in white.

Supplementary Figure S2

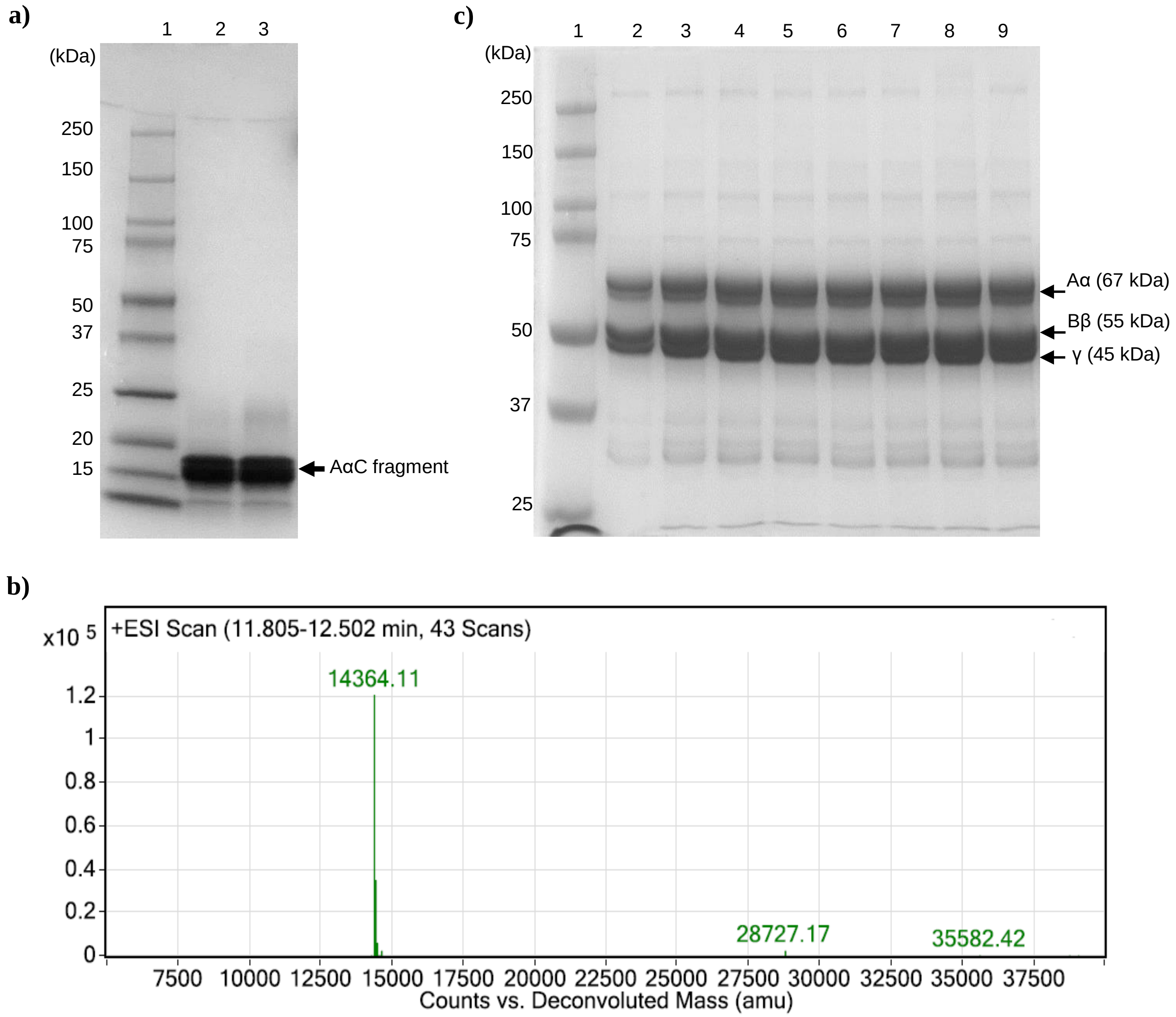

Figure S2. a) SDS-PAGE analysis of non-oxidized AαC fragment (AαC 419 - 502) (lane 2) and 150 µM HOCl oxidized AαC fragment (AαC 419 - 502) (lane 3) under non-reduced condition. Protein marker was loaded into lane 1. The non-oxidized AαC fragment was used as a control. **b**) Molecular mass determination of AαC fragment was analyzed by ESI-TOF mass spectrometry. The mass range from 7250 to 40000 Da was analyzed. The peak with the highest intensity in the mass spectrum indicated the measured molecular mass of AαC fragment. **c**) SDS-PAGE analysis of fibrinogen control (0 HOCl µM) and the oxidized fibrinogen solutions under reduced condition. The purified fibrinogen was oxidized by increasing HOCl concentrations. Lane 1: protein marker, lane 2: control fibrinogen (0 µM HOCl), lane 3 - 9: fibrinogen solutions oxidized by increasing HOCl concentrations (10, 25, 50, 75, 100, 125, 150 µM). Fibrinogen without HOCl oxidation (0 µM HOCl) was used as a control. Full-length SDS-PAGE gels were presented.

Supplementary Figure S3

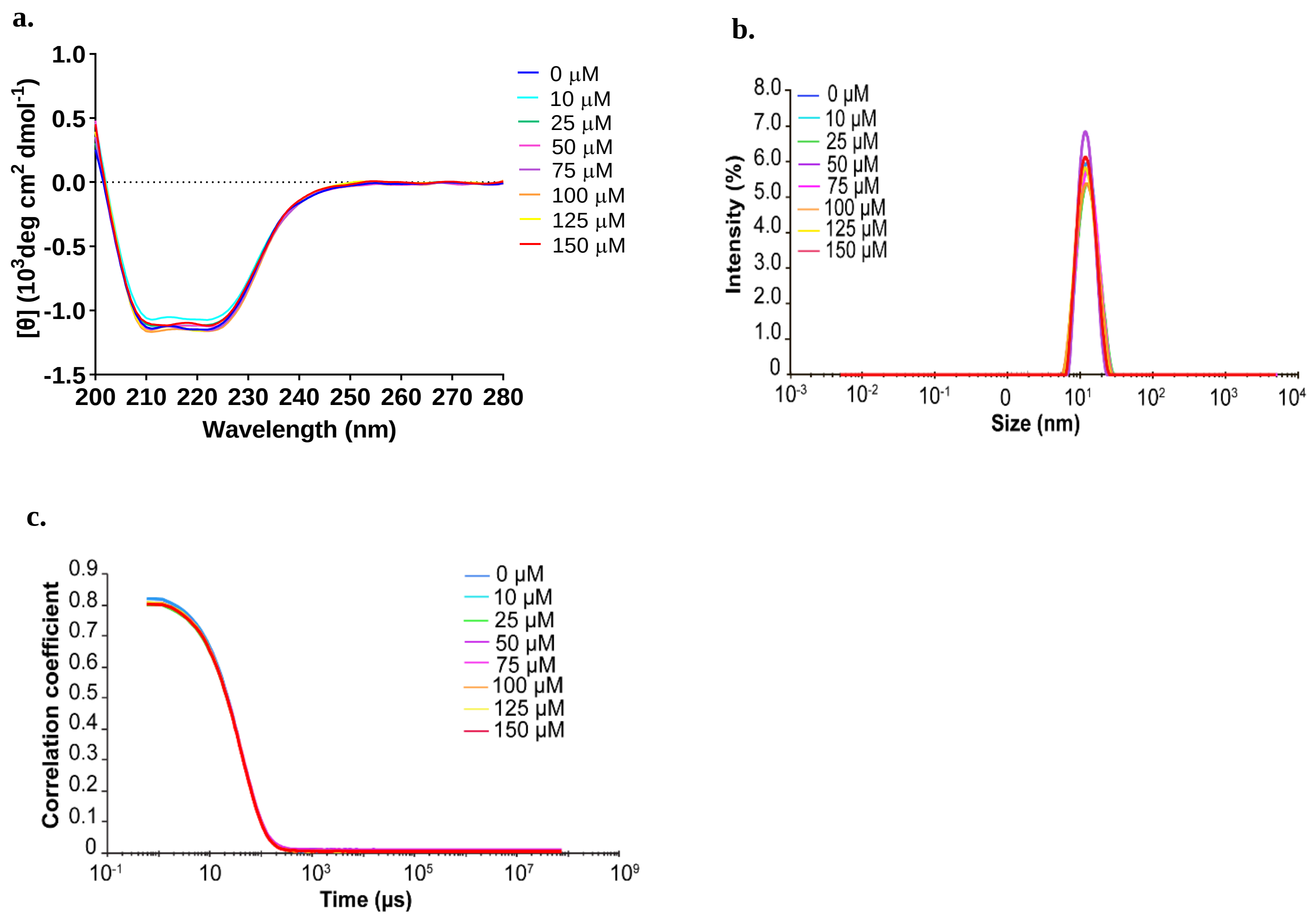

Figure S3. a) CD analysis of fibrinogen control (0 µM HOCl) and fibrinogen oxidized with increasing HOCl concentrations (10, 25, 50, 75, 100, 125, 150 µM). The acquired CD spectra were analyzed by the secondary structure prediction program supplied with the spectropolarimeter. All CD data were expressed as the mean residue ellipticity [θ], in units of degrees square centimeter per decimole. **b**) Hydrodynamic radius distribution by intensity for fibrinogen control and fibrinogen oxidized with increasing HOCl concentrations (10, 25, 50, 75, 100, 125, 150 µM) analyzed by DLS. **c**) Correlation coefficient decay curves of fibrinogen control and oxidized fibrinogen with increasing HOCl concentrations. Fibrinogen without oxidation (0 µM HOCl) was used as a control.

Supplementary Figure S4

a)

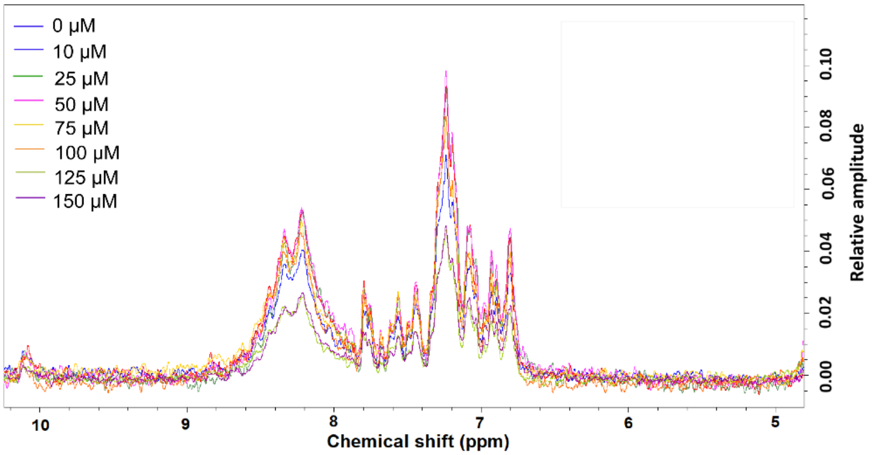

b)

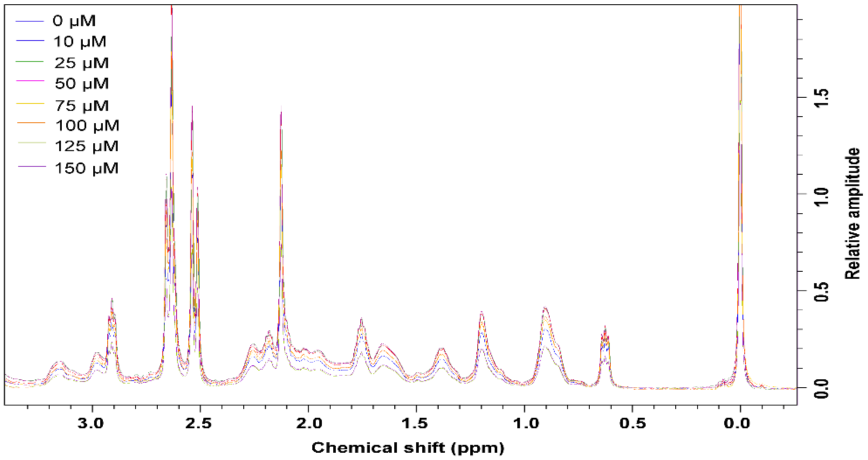

c)

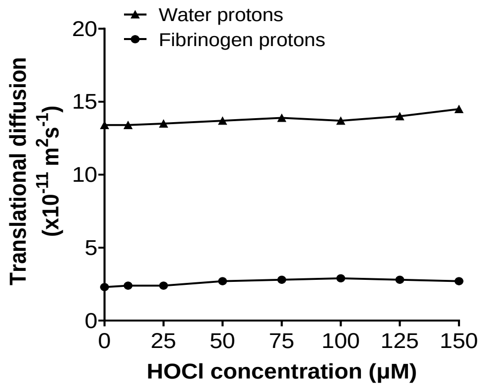

Figure S4. ^{1}H 1D NMR spectra of fibrinogen control (0 µM HOCl) and fibrinogen oxidized with increasing HOCl concentrations (10, 25, 50, 75, 100, 125, 150 µM). **a**) The ^{1}H signals of fibrinogen solutions were found at the amide region from 6.5 to 8.5 ppm. **b**) The fibrinogen methyl protons were observed at aliphatic region from 0.7 to 0.9 ppm. DSS (2,2-dimethyl-2-silapentane-5-sulfonic acid) was used as a chemical shift standard reference. The ^{1}H of DSS exhibited resonances at 2.91 ppm, 1.75 ppm, and 0.63 ppm with intensities lower than the reference resonance at 0 ppm. **c**) DOSY NMR analysis of diffusion coefficient (D) for fibrinogen and bound water protons in the fibrinogen solutions oxidized with increasing HOCl concentrations (0, 10, 25, 50, 75, 100, 125, 150 µM). Fibrinogen solution without oxidation (0 µM HOCl) was used as a control. The averaged ^{1}H fibrinogen signals (0.7 – 0.9 ppm) and water proton (4.72 ppm) were subjected for the analysis. The acquired data from the spectra were fitted to Stejskal-Tanner equation and diffusion coefficient of the samples were analyzed by NMRgenerator and CARA.

Supplementary Table

Table S1. Diffusion coefficient and T_2 relaxation times of water protons in the purified fibrinogen solutions and fibrin gels oxidized by an increasing HOCl concentrations

	Fibrinogen solutions [a]		Fibrin gels [b]	
HOCl (μM)	Diffusion coefficient ($x10^{-9}$ m^2s^{-1})	T_2 (ms)	Diffusion coefficient ($x10^{-9}$ m^2s^{-1})	T_2 (ms)
0 [c]	4.64	256.6	4.64	211.5
25	4.64	210.5	4.23	335.0*
50	4.64	210.5	4.64	335.2*
75	4.64	210.5	4.64	335.2*
100	4.64	210.5	3.85	335.2*
150	4.04	187.4	3.51	377.5*

[a] = Diffusion coefficient (D), and T_2 relaxation times of bulk water protons in the purified fibrinogen solutions and fibrinogen oxidized by an increasing concentration of HOCl (0, 25, 50, 75, 100, 150 μM HOCl); [b] = Diffusion coefficient and T_2 relaxation times of bulk water protons in the fibrin gels oxidized by an increasing concentration of HOCl (0, 25, 50, 75, 100, 150 μM HOCl). [c] = 0 μM HOCl fibrinogen solution and fibrin gel were used as their respective controls. * = Significant difference vs control at ≥ 1.5-fold change.

Table S2. Diffusion coefficient, T_1, and T_2 relaxation times of water protons in the plasma fibrinogen solutions and plasma fibrin gels formed by adding with an increasing % HOCl-oxidized fibrinogen

	Plasma fibrinogen solutions [a]			Plasma fibrin gels [b]		
% HOCl-oxidized fibrinogen [c]	Diffusion coefficient ($x10^{-9}$ m^2s^{-1})	T_1 (ms)	T_2 (ms)	Diffusion coefficient ($x10^{-9}$ m^2s^{-1})	T_1 (ms)	T_2 (ms)
0 [d]	3.85	2154	104.8	3.20	1918	148.5
10	3.85	2154	117	3.20	1918	166.8
20	3.85	2154	132.2	3.20	1918	187.4
30	4.23	2154	148.5	3.20	1918	187.4
40	4.23	2154	148.5	3.20	1918	236.4*
50	4.23	1918	236.4*	3.51	2420	335.3*

[a] = Diffusion coefficient (D), T_1, and T_2 relaxation times of bulk water protons in the plasma fibrinogen solutions added with an increasing % of HOCl-oxidized fibrinogen solution (10, 20, 30, 40, 50%); [b] = Diffusion coefficient (D), T_1, and T_2 relaxation times water protons in the plasma fibrin gel formed by an increasing % HOCl-oxidized fibrinogen solution; [c] = Purified fibrinogen solution was oxidized by 150 μM HOCl and added into plasma fibrinogen solution prior to clotting; [d] = 0% HOCl-oxidized fibrinogen solution and plasma fibrin gel were used as their respective controls. * = Significant difference vs control at ≥ 1.5-fold change.

Table S3. Fast acquisition of T_1 and T_2 relaxation times of water protons in the plasma fibrinogen solutions and plasma fibrin gels formed by adding with an increasing % HOCl-oxidized fibrinogens

	Plasma fibrinogen solutions [a]		Plasma fibrin gels [b]		
% HOCl-oxidized fibrinogen [c]	T_1 (ms)	T_2 (ms)	T_1 (ms)	T_{2i} (ms) [d]	T_2 (ms) [e]
0 [f]	2656	46.4	2656	25.6	187.4
10	2656	53.4	2656	30.5	215.4
20	2656	61.4	2656	35.1	284.8*
30	2656	61.4	2656	40.4*	284.8*
40	2656	70.6*	3054	61.4*	-
50	2656	123.3*	3054	81.1*	-

[a] = T_1 and T_2 relaxation times of bulk water protons in plasma fibrinogen solutions added with an increasing % of HOCl-oxidized fibrinogen (10, 20, 30, 40, 50%); [b] = T_1 and T_2 relaxation times of water protons in the plasma fibrin gels formed by an increasing % HOCl-oxidized fibrinogen solution; [c] = Purified fibrinogen was oxidized by 150 μM HOCl and added into plasma fibrinogen solution prior to clotting; [d] = T_{2i} relaxation time of intermediate water; [e] = T_2 relaxation time of bulk water; [f] = 0% HOCl-oxidized fibrinogen solution and plasma fibrin gel were used as their respective controls. * = Significant difference vs control at ≥ 1.5-fold change. The T_1/T_2 correlations were measured by 2D-NMR with fast acquisition.
